# Evaluation of Cannulated Compression Headless Screw (CCHS) as an alternative implant in comparison to standard S1-S2 screw fixation of the posterior pelvis ring: a biomechanical study

**DOI:** 10.1186/s12891-023-06312-1

**Published:** 2023-03-23

**Authors:** Till Berk, Ivan Zderic, Peter Varga, Peter Schwarzenberg, Felix Lesche, Sascha Halvachizadeh, Geoff Richards, Boyko Gueorguiev, Hans-Christoph Pape

**Affiliations:** 1 AO Research Institute Davos, Clavadelerstrasse 8, 7270 Davos, Switzerland; 2 Department of Trauma, University Hospital Zurich, Raemistrasse 100, 8091 Zurich, Switzerland; 3 Department of Gynecology and Obstetrics, Asklepios Clinic Wandsbek, Alphonsstraße 14, 22043 Hamburg, Germany; 4 University of Zurich, Harald-Tscherne Laboratory for Orthopedic and Trauma Research, Sternwartstrasse 14, 8091 Zurich, Switzerland

**Keywords:** Posterior pelvis ring injuries, Sacroiliac joint disruption, Cannulated Compression Headless Screw, Biomechanics

## Abstract

**Background/Purpose:**

Posterior pelvis ring injuries represent typical high-energy trauma injuries in young adults. Joint stabilization with two cannulated sacroiliac (SI) screws at the level of sacral vertebrae S1 and S2 is a well-established procedure. However, high failure- and implant removal (IR) rates have been reported. Especially, the washer recovery can pose the most difficult part of the IR surgery, which is often associated with complications.

The aim of this biomechanical study was to evaluate the stability of S1-S2 fixation of the SI joint using three different screw designs.

**Methods:**

Eighteen artificial hemi-pelvises were assigned to three groups (*n* = 6) for SI joint stabilization through S1 and S2 corridors using either two 7.5 mm cannulated compression headless screws (group CCH), two 7.3 mm partially threaded SI screws (group PT), or two 7.3 mm fully threaded SI screws (group FT). An SI joint dislocation injury type III APC according to the Young and Burgess classification was simulated before implantation. All specimens were biomechanically tested to failure in upright standing position under progressively increasing cyclic loading. Interfragmentary and bone-implant movements were captured via motion tracking and evaluated at four time points between 4000 and 7000 cycles.

**Results:**

Combined interfragmentary angular displacement movements in coronal and transverse plane between ilium and sacrum, evaluated over the measured four time points, were significantly bigger in group FT versus both groups CCH and PT, *p* ≤ 0.047. In addition, angular displacement of the screw axis within the ilium under consideration of both these planes was significantly bigger in group FT versus group PT, *p* = 0.038. However, no significant differences were observed among the groups for screw tip cutout movements in the sacrum, *p* = 0.321. Cycles to failure were highest in group PT (9885 ± 1712), followed by group CCH (9820 ± 597), and group FT (7202 ± 1087), being significantly lower in group FT compared to both groups CCH and PT, *p* ≤ 0.027.

**Conclusion:**

From a biomechanical perspective, S1-S2 SI joint fixation using two cannulated compression headless screws or two partially threaded SI screws exhibited better interfragmentary stability compared to two fully threaded SI screws. The former can therefore be considered as a valid alternative to standard SI screw fixation in posterior pelvis ring injuries. In addition, partially threaded screw fixation was associated with less bone-implant movements versus fully threaded screw fixation. Further human cadaveric biomechanical studies with larger sample size should be initiated to understand better the potential of cannulated compression headless screw fixation for the therapy of the injured posterior pelvis ring in young trauma patients.

## Introduction

High-energy pelvis trauma prevails in younger adults as opposed to low-energy trauma in elderly patients with poor bone quality associated with high morbidity and mortality rates [1]. Biomechanical studies have reported that the posterior sacroiliac (SI) complex provides more stability (60%) of the pelvis ring than the anterior structures (40%) [2]. Therefore, a stable restoration of the posterior pelvis ring is of utmost importance to recover initial stability. The posttraumatic stabilization of posterior pelvis ring injuries can be performed with various fixation techniques [3–5]. Surgical stabilization of vertically unstable posterior pelvis ring injuries via a fixation through the corridor of S1 and S2 vertebral bodies using two SI screws is the standard of care [6, 7]. Percutaneously inserted SI screws have become the surgical gold standard for fixation of fractures through the SI joint or sacrum [8, 9]. However, implant failures using S1-S2 SI screws are reported by most investigators [10]. It was concluded that in 57% of the follow-up cohort, implant removal (IR) was indicated after SI screw fixation, and that IR is justified in young patients [11]. Publications concerning complications regarding IR report rates of 3–20% [12–14]. The recovery of the washer can be the most difficult part of the procedure and may be the most time consuming aspect of  an IR surgery. It has been described as challenging and as the aspect being most prone to problems [15, 16]. Considering these adverse observations, alternative screw designs avoiding the use of a washer could be of benefit. Cannulated compression headless screws (CCHS) have been introduced as a dependable choice of internal fracture fixation in recent years [17]. They are well-established and commonly used in scaphoid fractures, metacarpal fractures, tibia fractures and femoral neck fractures [18–21]. For young patients with potentially good bone quality, countersunk screws could possibly provide comparable stability with possibly lower reoperation rates due to less or undemanding IR surgeries in the future. To our knowledge, the surgical technique for CCHS fixation of the posterior pelvis ring has not been evaluated in the literature so far.

Therefore, the aim of this biomechanical study was to evaluate the stability of S1-S2 fixation of the SI joint with CCHS versus conventional fully and partially threaded SI screws in an artificial pelvic bone model. It was hypothesized that CCHS would provide equivalent stability as conventional SI screws.

## Materials and methods

Nine anatomical composite pelvises (Model 4060®, Synbone, Zizers, Switzerland) were used. A posterior SI joint dislocation injury type III APC according to the Young and Burgess classification was simulated on each side of all pelvises [22]. For that purpose, the specimens were manipulated by removing the stabilization material of the symphysis and the SI joint. This led to a pubic symphysis diastasis larger than 5 cm, with an ipsilateral anterior SI joint diastasis, and hence a widening of the SI joint anteriorly and posteriorly, resulting in a global instability.

Each pelvis was considered for instrumentation and testing of its left and right site, resulting in eighteen available hemi-pelvis constructs, which were assigned to three groups of six specimens each (*n* = 6) for instrumentation as follows:

- Group CCH: Stabilization of the posterior pelvis ring with two 7.5 mmlong-threaded CCHS, 90 mm in length for S1 and 65 mm in length for S2;
- Group PT: Stabilization of the posterior pelvis ring with two 7.3 mm partially threaded cannulated SI screws and washers, 90 mm in length for S1 and 65 mm in length for S2;
- Group FT: Stabilization of the posterior pelvis ring with two 7.3 mm fully threaded cannulated SI screws and washers, 90 mm in length for S1 and 65 mm in length for S2.

The screws in groups PT and FT were made of stainless steel (316LVM), whereas the screws in group CCH were made of titanium alloy (Ti-6Al-4V ELI). All implants were provided by the same manufacturer (DePuy Synthes, Zuchwil, Switzerland). A hemi-pelvis model was considered for biomechanical testing; therefore, both sides of the same pelvis were instrumented. The symphysis was discontinued by cutting the rami at midshaft on both sides. The ilium of the non-tested site was detached until the testing of the contralateral site was completed. Afterwards, the contralateral side was instrumented and the ilium of the tested side detached. The screw lengths were chosen such that their tips did not cross the midline of the sacrum, thus allowing for bilateral testing. Each group consisted of specimens with three left and three right SI joint fixations. The surgical treatment of the APC III injury was carried out according to the AO principles [23]. An experienced surgeon with senior consultant status performed all procedures.

### Surgical procedure

**Fig. 1:**
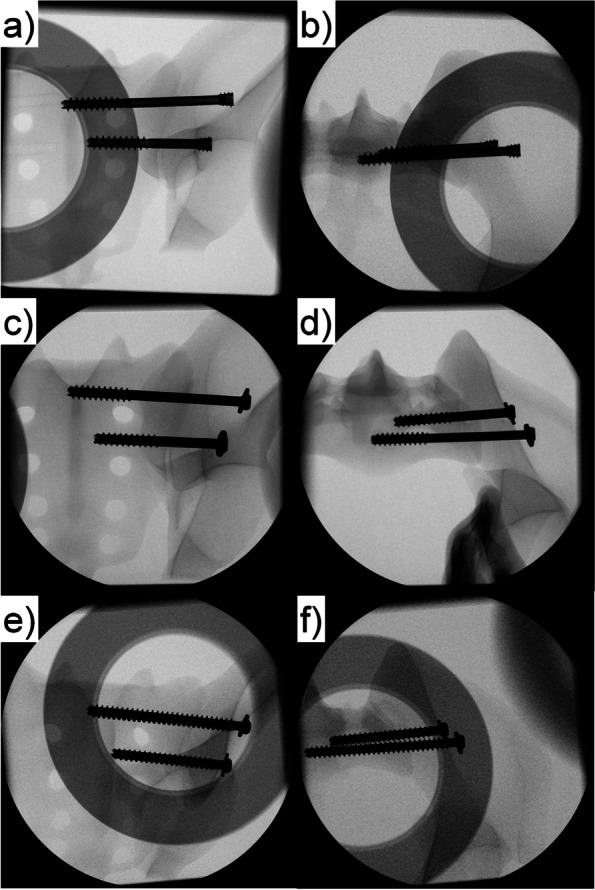
X-rays after instrumentation visualizing exemplified specimens from group CCH (**a**, **b**), PT (**c**, **d**) and FT (**e**, **f**) in inlet (**a**, **c**, **e**) and outlet (**b**, **d**, **f**) views

Following anatomical reduction of the SI joint, in all groups the screws were placed perpendicular to it within the bone specimen under avoidance of the nerve root tunnels using guidewires. All screws were placed with the help of a custom aiming device for standardized and repeatable wire- and screw placement. This approach assured avoidance of any perforations, via falsae, or cortical disruptions. The S1 screw was always placed first, followed by insertion of the S2 one. The screws just extended to the midline of the sacral body and  were tightened according to the best of the operator's knowledge. After instrumentation, true lateral, inlet and outlet projections were visualized via X-rays for documentation and verification of the positioning of the screws (Fig. 1). All surgical procedures were performed strictly following the AO surgery references and recommendations [23].

### Biomechanical testing

**Fig. 2:**
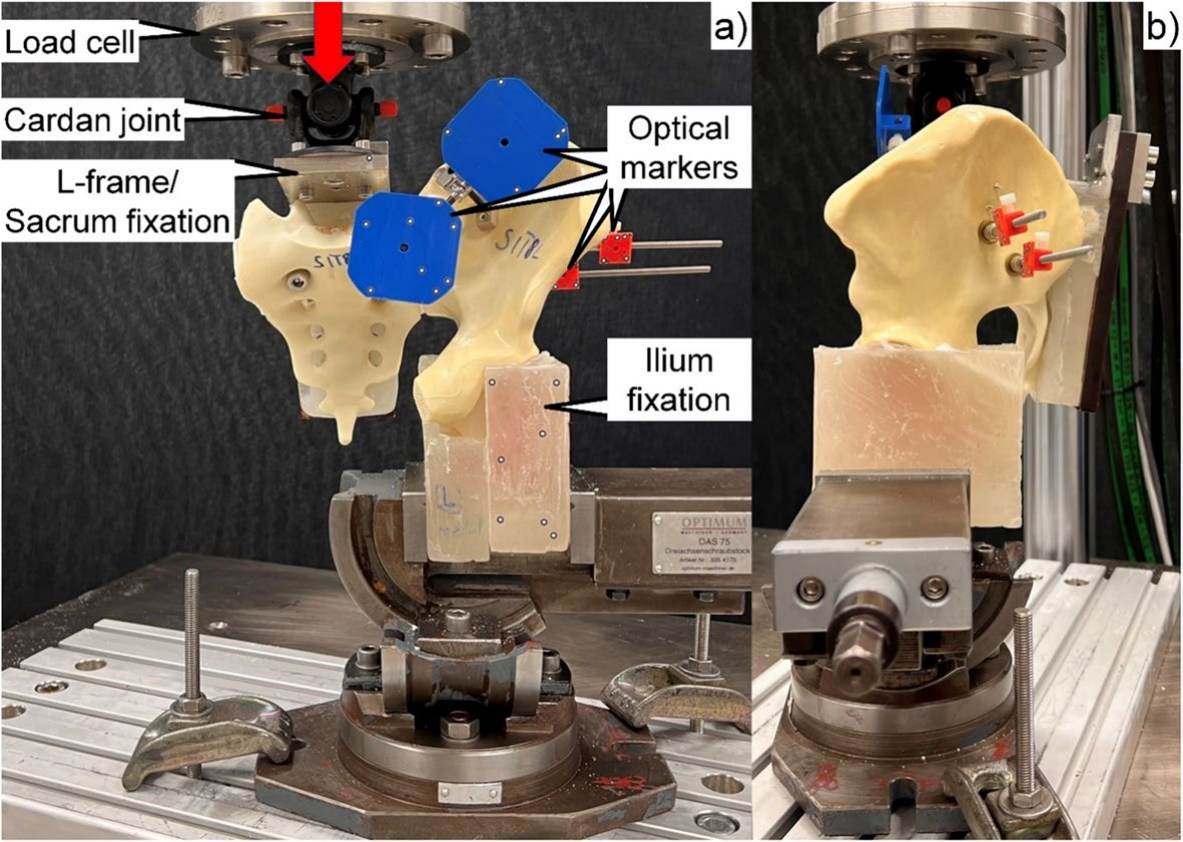
Setup with a specimen mounted for biomechanical testing. Vertical arrow denotes loading direction

Biomechanical testing was performed on a servohydraulic material test system (Bionix 858.20; MTS Systems, Eden Prairie, MN, USA) equipped with a 5 kN/50 Nm load cell. The setup with a specimen mounted for testing is presented in Fig. 2. Each hemi-pelvis specimen was aligned and tested in an upright standing position. The ilium of the tested side was constrained to the machine base via a vice fixing the portion of the ilium between the ramus and the proximal border of the acetabulum. Two custom polymethylmethacrylate (PMMA, Suter Kunststoffe AG, Fraubrunnen, Switzerland) blocks were molded for repeated use, allowing for a homogeneous clamping force exerted by the vice on the corresponding bony region. Loading along the machine axis was applied to the sacrum with 41 mm anterior offset relative to the posterior-superior S1 endplate aspect, generating the required bending moment around the medio-lateral axis as calculated from a previous inverse-dynamic gait analysis [24]. For that purpose, a custom steel plate with an L-profile was used. The sacrum was fixed to the vertically oriented side of the plate with two screws inserted through the fourth sacral foramen. The horizontal part of the plate was attached to the machine actuator via a cardan joint. Four retro-reflective marker sets were attached to the iliac crest, the sacrum, and the two SI screws for optical motion tracking.

The loading protocol commenced with an initial nondestructive quasi-static ramp from 20 N preload to 200 N at a rate of 18 N/s, followed by progressively increasing cyclic loading in axial compression with a physiological profile of each cycle at a rate of 2 Hz [25]. Keeping the valley load at a constant level of 20 N, the peak load, starting at 200 N, was monotonically increased cycle by cycle at a rate of 0.05 N/cycle until catastrophic failure of the specimen [26, 27].

### Data acquisition & analysis

Machine data in terms of axial displacement and axial load were acquired at 200 Hz from the machine transducer and load cell throughout the tests. Based on these, Initial Construct Stiffness was calculated from the ascending load–displacement curve of the initial quasi-static ramp.

The positions of the optical markers attached to the sacrum, ilium, and the two SI screws were recorded at 20 Hz from a stereographic camera system (Aramis SRX, Carl Zeiss GOM Metrology GmbH, Braunschweig, Germany) for motion tracking. Based on these, interfragmentary and bone-implant movements were analyzed in all six degrees of freedom. Total Displacement was defined as the magnitude of the deviating movement between the two fragments measured in the most superior fracture aspect. Gap Angle was defined as the combined angular displacement in sagittal and coronal plane of the sacrum relative to the ilium. Furthermore, Screw Tilt Ilium was defined as the angular displacement of the S1 screw relative to the ilium. Next, Screw Tip Cutout was defined as the displacement of the S1 screw tip relative to the sacrum perpendicular to the screw axis. The outcome measures were evaluated at four time points of cyclic testing after 4000, 5000, 6000, and 7000 cycles. The latter represented the highest rounded number of cycles when none of the specimens had failed. The outcome measures were considered with respect to their values at the beginning of the cyclic test and were calculated under peak loading condition. In addition, a clinically relevant criterion for specimen failure was arbitrarily set at 5° Gap Angle, and the number of cycles until its fulfillment – defined as Cycles To Failure – was evaluated together with the corresponding peak load – defined as Load At Failure.

Statistical analysis was performed with SPSS software package (v.27, IBM SPSS, Armonk, NY, USA). Normality of data distribution was screened and proved with Shapiro-Wilk test. Mean value and standard deviation (SD) were calculated for each parameter of interest and group separately. One-Way Analysis of Variance (ANOVA) and General Linear Model Repeated Measures tests with Bonferroni post-hoc test for multiple comparisons were conducted to detect significant differences among the treatment groups. Level of significance was set at 0.05 for all statistical tests.

## Results

Initial Construct Stiffness was 92.1 ± 29.1 N/mm for group CCH, 85.3 ± 20.2 N/mm for group PT, and 60.0 ± 14.9 N/mm for group FT, without significant differences among the groups, *p* = 0.137.

The outcome measures evaluated after 4000, 5000, 6000, and 7000 cycles are summarized in Table 1. Gap Angle was associated with significantly bigger values in group FT compared to both groups CCH and PT, *p* ≤ 0.047. Similarly, instrumentation with fully threaded screws in group FT resulted in significantly bigger Screw Tilt Ilium compared to partially threaded screws in group PT, *p* = 0.038, without further significant differences among the groups, *p* ≥ 0.118. Finally, both Total Displacement and Screw Tip Cutout remained not significantly different among the groups, *p* ≥ 0.200.

**Table 1:** Outcome measures evaluated after 4000, 5000, 6000, and 7000 cycles, presented for each treatment group in terms of mean and SD together with *p*-values from the statistical evaluation over cycles and among the groups

Parameter of Interest	Treatment group	Cycles				*p*-value among groups		
		**4000**	**5000**	**6000**	**7000**	CCH	PT	FT
**Total Displacement (mm)**	CCH	1.63 (1.03)	2.30 (1.19)	2.79 (1.20)	3.90 (1.15)	0.200		
	PT	1.55 (1.11)	2.31 (1.18)	2.96 (1.50)	4.00 (1.83)			
	FT	2.13 (1.44)	2.84 (1.77)	4.44 (0.98)	6.75 (0.44)			
	***p*-value over cycles**	CCH: *p* < 0.001; PT: *p* = 0.011; FT: *p* < 0.001				-		
**Gap Angle (°)**	CCH	1.29 (0.76)	1.79 (0.77)	2.07 (0.79)	2.43 (0.86)	-	0.673	0.047
	PT	0.86 (0.57)	1.07 (0.70)	1.34 (0.82)	1.64 (0.97)	0.673	-	0.006
	FT	1.75 (0.76)	2.30 (1.00)	3.58 (0.54)	6.24 (2.58)	0.047	0.006	-
	***p*-value over cycles**	CCH: *p* = 0.004; PT: *p* = 0.017; FT: *p* = 0.047				-		
**Screw Tilt Ilium (°)**	CCH	1.66 (1.04)	2.35 (1.16)	2.89 (1.18)	3.52 (1.20)	-	> 0.999	0.118
	PT	1.36 (0.64)	1.90 (0.73)	2.45 (0.86)	2.92 (1.02)	> 0.999	-	0.038
	FT	2.31 (1.20)	3.12 (1.57)	4.65 (0.84)	6.86 (1.46)	0.118	0.038	-
	***p*-value over cycles**	CCH: *p* < 0.001; PT: *p* = 0.001; FT: *p* < 0.001				-		
**Screw Tip Cutout (mm)**	CCH	0.22 (0.21)	0.26 (0.17)	0.43 (0.23)	0.56 (0.36)	0.321		
	PT	0.20 (0.07)	0.29 (0.16)	0.43 (0.37)	0.66 (0.40)			
	FT	0.46 (0.13)	0.46 (0.25)	0.65 (0.30)	0.76 (0.41)			
	***p*-value over cycles**	CCH: *p* = 0.072; PT: *p* = 0.024; FT: *p* = 0.135				-		

**Fig. 3:**
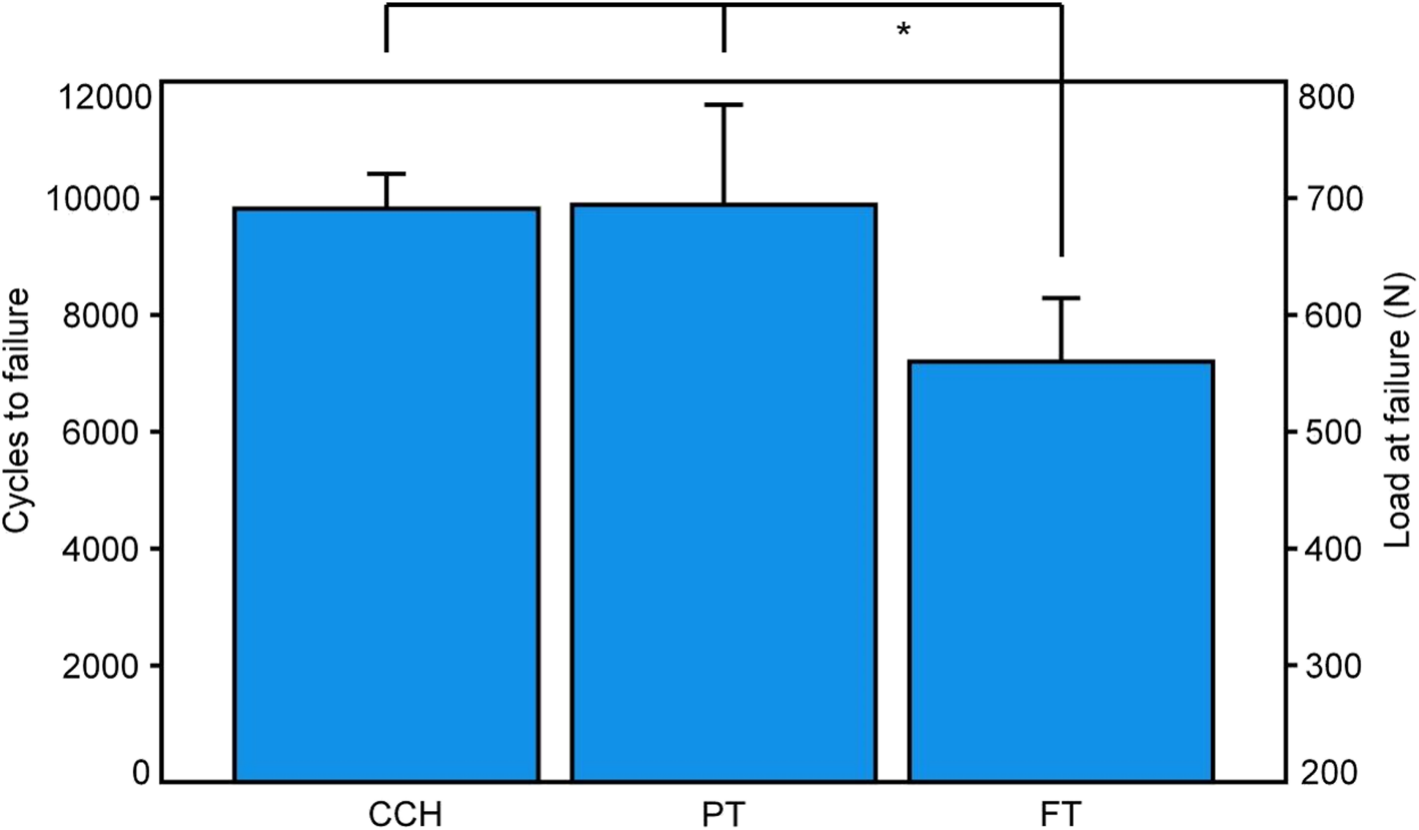
Cycles To Failure and corresponding Load At Failure , presented for each group separately in terms of mean and SD. Star indicates significant differences between the groups

Cycles To Failure and the corresponding Load At Failure were highest in group PT (9885 ± 1712/694.3 ± 85.6 N), followed by group CCH (9820 ± 597/691.0 ± 29.9 N), and group FT (7202 ± 1087/560.1 ± 54.4 N), with significantly lower values in group FT compared to both groups CCH and PT, *p* ≤ 0.027 (Fig. 3). The failure modes were predominantly expressed by fracturing of the ilium between the ilium fixation and the second SI screw.

## Discussion

The objective of this study was to assess the biomechanical potential of cannulated compression headless screws used for fixation of posterior pelvis ring injuries, and consecutively, to possibly decrease the rate of implant failures, consolidate minimally invasive procedures, and avoid secondary implant removal surgeries.

With regard to the number of test cycles until failure and the corresponding failure load, both groups CCH and PT demonstrated considerably better stability versus group FT, and comparable stability versus each other. On the other hand, all three groups revealed similar initial construct stiffness.

SI screw fixation of the posterior pelvis ring in unstable injuries is a well-established surgical procedure. SI screw fixation through S1 and S2 sacral body corridors was described to feature the best implant positioning [10]. Therefore, such screw placements were selected in the current study. The fixation methods in the current treatment groups are frequently used as a standard in the clinical practice [28, 29]. Previous work could not demonstrate significant differences between the different types of fixation [28, 30, 31].

It is known that the SI joint has a physiological movement from 2 mm to 4 mm [32–34]. The fully threaded screws in group FT could have provided an overly rigid structure impeding the physiological movement of the SI joint. This could have therefore led to loosening of the screws and consequently to the reduced stability in group FT. Another possible explanation for the inferior performance of this group could be that the fully threaded screws were not able to generate the same compression force within the SI joint, which could have led to increased movements and therefore to higher loosening rates.

For the partially threaded screws in groups PT and CCH, a movement of the joint around the threadless smooth part of the screw shafts is conceivable and could therefore explain their higher stability during testing. Likewise, as seen in fixations of ankle fractures, partially threaded screw designs have been proven to decrease both initial screw stiffness and yield load, compared to fully threaded screws [35]. Generally, the mobility should be minimized after insertion of two screws. The higher interfragmentary stability of the fixations with partially treaded versus fully threaded screws, indicated by Gap Angle, was also evidenced by Total Displacement (at the most superior aspect of the fracture line), however, the latter evidence could not be substantiated statistically due to the fact that the differences between the groups became pronounced at a later stage of cyclic testing compared to Gap Angle.

The hypothetical target group for SI screw fixation with cannulated compression headless screws considers young patients with good bone quality. This study was able to show a partly superior and partly comparable stability of fixation with these screws versus conventional SI screws.

There is very scarce literature on IR rates following SI fixation, with most of the studies discussing indications for IR regarding the anterior pelvis ring [36, 37].

A complete IR may be necessary in the event of suspected infection or metal allergies, as well as in case of unexplained pain and/or explicit patient request. The aspect of removing the washer – "the Washer problem" – has already been described in the literature [15, 16]. To combat these issues, an endoscopic SI IR approach was introduced in order to minimalize radiation exposure and soft tissue trauma [38]. An advantage of the surgical treatment with CCHS is its comparable stability without using a washer. As a result, the surgery times could be significantly shorter when IR is necessary.

A previous biomechanical investigation reported that 7.3 mm fully threaded transiliac–transsacral screw fixation would result in less fracture displacement at the S1 foramen compared to 7.3 mm partially threaded transiliac–transsacral screw fixation in vertically transforaminal sacral fractures [39]. This result is controversial to the findings from the current study because group FT was related with inferior outcomes. The controversy could be explained with the transsacral approach using an additional anchorage in the contralateral cortex. Hence, higher compression could have been exerted at the fracture site, leading to less fracture displacement. On the other hand, in group FT the tip of the screw was located in cancellous bone, which does not provide any additional support for compression. In the treatment of a unilateral injury of the SI joint in young patients, the authors of this study consider it debatable to modify a healthy SI joint on the contralateral side via a screw placement. Naturally, there are cases when, due to massive instability, the surgeon has no other choice. However, this should always be carefully considered in young patients. Furthermore, the referred study investigated a different pattern of injury.

A recent study with similarly used specimens concluded that fully threaded 7.3 mm screws could withstand significantly higher axial loads to failure than partially threaded 7.3 mm screws [40]. However, the created constructs were not intended to be representative of the SI joint. When these findings are compared with the results from our study, it becomes clear that the SI joint is biomechanically of particular importance and plays a significant part in the surgical treatment strategy. The ideal surgical technique for SI or transiliac–transsacral screw fixation remains controversial due to the diversity of posterior pelvis fracture morphologies and considerable forces transmitted through the SI joint [10, 41, 42]. Supplementary studies in this field of interest are therefore beneficial for a better understanding of this complex topic. The current study was able to present a possible alternative implant for pelvis surgery, which indicates partly superior and partly comparable stability compared to the standard treatment. In addition, all implants described in this investigation can be inserted minimally invasively since percutaneous SI screw fixation is known for reduced blood loss and shorter surgery times [43, 44].

Further studies should follow in order to consolidate the obtained results on real bones and, if applicable, to confirm them in a clinical study.

### Strength & limitations

The main limitation of this study is the chosen specimen model implicating less physiological conditions due to lack of ligaments. This makes the simulation of a ligamentous injury to the pelvis difficult. However, as this is an experimental study with an innovative approach, with no available data for this approach in the literature, and unpredictable outcomes, the authors have chosen the above-mentioned specimen model as a first step. Only after such a first step it is ethically justifiable to carry out a cadaveric study as a second step. Further, to accommodate the main limitation, a type III APC injury according to the Young and Burgess classification was deliberately chosen. According to its definition, the ligaments are ruptured and an absolutely unstable situation prevails. Therefore, the ligaments could be largely neglected in the selected test series. Nevertheless, it is known that artificial pelvises allow for a standardization of the treatment groups – thus overpowering the almost uncountable variations in bone quality seen in human cadaveric specimens – and are more cost-effective [45–48]. Synthetic bone specimens are frequently and effectively used in various biomechanical studies, explicitly related to the pelvis [31, 45, 49–51]. Additionally, the poor availability of cadavers can limit the sample size for biomechanical experimentations and it is known that the sample size in previous publications is generally small [52]. Use of artificial bone models minimizes the variability of test results between tested groups of specimens [31, 53].

Additionally, the chosen sample size was relatively small, yet comparable to similar biomechanical studies investigating posterior pelvis fixation techniques [31, 49–51, 54]. Another point for discussion is the design of the used screws. The screws in the CCH group were 0.2 mm bigger in diameter than the screws used in the comparison groups (7.5 mm CCHS versus 7.3 mm cannulated screws in groups PT and FT). All screws were inserted with the help of a custom aiming device for standardized and constantly identical wire/screw placement. Since this approach did not lead to any difficulties during screw placements – such as perforation, via falsa, or cortical disruption – we believe that this circumstance can be neglected at this stage of research. As thicker implants are known to be associated with higher stability, this could also be a potential explanation for the current results. However, the same applies to the materials of the implants, as a steel 7.3 mm screw is known to be more stable than a titanium 7.5 mm screw. Since the difference in diameter was only 0.2 mm, the authors assume this circumstance as negligible. Another limitation is the lack of insertion torque values, which were not investigated and that could have better highlighted possible differences between the fully threaded and partially threaded screws. However, since all screw fixations were performed by the same experienced surgeon, a homogeneous and comparable tightening of the screws can be assumed. Whether a CCHS is also suitable for osteoporotic bone in elderly patients needs to be investigated in future studies.

## Conclusion

From a biomechanical perspective, S1-S2 SI joint fixation using two cannulated compression headless screws or two partially threaded SI screws exhibited better interfragmentary stability compared to two fully threaded SI screws. The former can therefore be considered as a valid alternative to standard SI screw fixation in posterior pelvis ring injuries. In addition, partially threaded screw fixation was associated with less bone-implant movements versus fully threaded screw fixation. Further human cadaveric biomechanical studies with larger sample size should be initiated to understand better the potential of cannulated compression headless screw fixation for the therapy of the injured posterior pelvis ring in young trauma patients.

## Data Availability

The collected data will be stored securely in our institute for 10 years. During this period, they are still available upon request. After 10 years, the data will be deleted, however, all the datasets analyzed or generated during this study will be available from corresponding author upon reasonable request.
